# The impact of altered dietary adenine concentrations on the gut microbiota in *Drosophila*

**DOI:** 10.3389/fmicb.2024.1433155

**Published:** 2024-08-05

**Authors:** Xianglin Yin, Qing Tong, Jingtao Wang, Jinfeng Wei, Zhenbo Qin, Yujie Wu, Ruidi Zhang, Baosheng Guan, Hongbin Qiu

**Affiliations:** ^1^School of Basic Medical Sciences, Jiamusi University, Heilongjiang, China; ^2^School of Public Health, Jiamusi University, Heilongjiang, China; ^3^School of Biology and Agriculture, Jiamusi University, Heilongjiang, China

**Keywords:** adenine, gut microbiome, hyperuricaemia, metabolic disease, uric acid

## Abstract

The gut microbiota influences host metabolism and health, impacting diseases. Research into how diet affects gut microbiome dynamics in model organisms is crucial but underexplored. Herein, we examined how dietary adenine affects uric acid levels and the gut microbiota over five generations of *Drosophila melanogaster*. Wild-type W1118 flies consumed diets with various adenine concentrations (GC: 0%, GL: 0.05%, and GH: 0.10%), and their gut microbiota were assessed via Illumina MiSeq sequencing. Adenine intake significantly increased uric acid levels in the GH group > the GC group. Despite no significant differences in the alpha diversity indices, there were significant disparities in the gut microbiota health index (GMHI) and dysbiosis index (MDI) among the groups. Adenine concentrations significantly altered the diversity and composition of the gut microbiota. High adenine intake correlated with increased uric acid levels and microbial population shifts, notably affecting the abundances of Proteobacteria and Firmicutes. The gut microbiota phenotypes included mobile elements, gram-positive bacteria, biofilm-forming bacteria, and gram-negative bacteria. The significantly enriched KEGG pathways included ageing, carbohydrate metabolism, and the immune system. In conclusion, adenine intake increases uric acid levels, alters gut microbiota, and affects KEGG pathways in *Drosophila* across generations. This study highlights the impact of dietary adenine on uric acid levels and the gut microbiota, providing insights into intergenerational nutritional effects.

## 1 Introduction

The gut microbiota has a significant effect on animal health and metabolic diseases (Tong et al., 2020a). It not only aids in the digestion of complex carbohydrates that the host cannot breakdown to produce key nutrients such as short-chain fatty acids but also regulates the immune system and protects the host from pathogen invasion (Li et al., 2017; Ma et al., 2019). Moreover, these microbial communities influence energy balance and metabolic processes, playing a central role in overall health maintenance and disease prevention (Fontaine et al., 2018). The gut microbiota regulates the metabolic health of the host through various mechanisms, including energy harvesting, inflammatory responses, insulin sensitivity, weight management, and cholesterol and lipid metabolism (Nagpal et al., 2016; Nie et al., 2017). Changes in the gut microbiota are closely related to uric acid metabolic disorders such as hyperuricaemia (HUA), challenging the traditional concept that uric acid is excreted only through the kidneys (Liu et al., 2020; Wang J. et al., 2022). Therefore, a deep understanding of how the gut microbiota specifically interacts with dietary components, such as adenine, to influence uric acid levels can enhance our understanding of the mechanisms of health and disease and provide new avenues for the development of prevention and treatment strategies.

HUA poses a global health risk. Approximately 21% of adults in the United States are affected by HUA, with a 3.9% prevalence rate of gout (Han et al., 2023). Approximately 30% to 50% of HUA cases are attributed to dietary factors, including consuming high-purine foods, a high-sugar diet, excessive alcohol consumption and other dietary habits that directly influence uric acid levels (Wang J. et al., 2022). Recent scientific research has improved the understanding of the relationship between the gut microbiota and host health (Zhang et al., 2020). The gut is a major location for uric acid transport and elimination, although its metabolism is unknown (Wen et al., 2024). HUA may be caused by a gut microbial imbalance (Zhou et al., 2024). Alterations in the gut microbiota structure can cause metabolic problems, purine metabolism enzyme production, and inflammatory factor release, all of which are linked to HUA development (Zhou et al., 2024). These findings highlight the significant role of dietary habits, particularly purine and adenine intake, in influencing the gut microbiota composition and, subsequently, uric acid levels. Regulating the gut microbiota through a balanced diet, quitting smoking, limiting alcohol, moderate exercise, and probiotics can benefit HUA treatment (Luo et al., 2024; Sun et al., 2024). The gut microbiota has become a new target for exploring HUA pathogenesis, and the development of HUA treatments targeting the microbiota is receiving increasing attention (Wang Z. et al., 2022). Thus, understanding the interaction between the gut microbiota and HUA is crucial for disease prevention and treatment; however, the mechanisms by which dietary components affect the gut microbiome remain underexplored.

*Drosophila melanogaster*, owing to its biological simplicity and highly controllable experimental environment, has become an ideal model organism for the study of human diseases (Fischer et al., 2023). While *Drosophila* provide a unique and valuable perspective in genetics, developmental biology, and metabolic pathology (Rand et al., 2023), it is important to recognize these limitations when extrapolating findings to human conditions. Specific differences in physiology and microbiota composition can influence the translatability of research findings. In particular, in the study of metabolic diseases, *Drosophila* provide a unique perspective, especially in the study of pathological purine metabolism and pathological uric acid regulatory mechanisms (Yamauchi et al., 2020). In recent years, significant progress has been made in simulating human diseases via the *Drosophila* model (Pandey and Nichols, 2011). By adjusting the diet and gene expression of *Drosophila*, scientists can simulate the pathological metabolic state of humans and then observe changes in the microbial community and their potential impact on the metabolism of the host (Trinder et al., 2017). For example, in another study, a high-sugar diet was used to simulate high sugar intake by humans, and the effects of this diet on the composition of the *Drosophila* gut microbiota and the other impacts on fly health were observed (Zhang et al., 2017). In addition, research has shown that changes in the structure and function of the gut microbiota may directly affect uric acid production and excretion, providing a possible new approach for the treatment and prevention of HUA and gout (Wang Z. et al., 2022). However, there have not yet been studies on the impact of long-term oral intake of adenine in adult *Drosophila*, indicating that further research is needed in this field.

In this study, we used *Drosophila* as a model organism to investigate the impact of different dietary adenine concentrations on uric acid levels and the gut microbiota composition, as well as the downstream biological effects (Erkosar et al., 2023). We assessed the impact of dietary adenine on the gut microbiota across five *Drosophila* generations (Dodge et al., 2023). Examining the gut microbiota across multiple *Drosophila* generations revealed the stability of these communities, their role in *Drosophila* physiology and ecology, and the specific effects of dietary adenine, enhancing our understanding of the *Drosophila* ecosystem (Dodge et al., 2023; Tafesh-Edwards and Eleftherianos, 2023). Furthermore, analyzing multigeneration data provides a comprehensive view of the impact of adenine on *Drosophila*, providing detailed insights for related research (Bhattacharya et al., 2023). Our hypothesis is that variations in dietary purine concentrations, especially uric acid levels, alter the gut microbial community and affect metabolic health. This study uniquely examined the effects of dietary adenine on the Drosophila gut microbiota across five generations (Dodge et al., 2023), revealing community stability and its physiological and ecological roles and providing detailed insights into multigenerational impacts (Bhattacharya et al., 2023).

## 2 Materials and methods

### 2.1 Experimental design and passage culture method

In this study, freshly hatched wild-type *D. melanogaster* (W1118) eggs were divided into three experimental groups: a control group fed on standard cornmeal medium, a low adenine group (0.05% adenine), and a high adenine group (0.1% adenine) (Baosheng et al., 2024). Following eclosion, the adults were identified within an eight-hour period, sex separated, and cultured for 15 days before specimen collection (Lang et al., 2019; Fuse et al., 2023; Baosheng et al., 2024). Each group, comprising fifty males and fifty females 2–3 days posteclosion, was then paired for breeding. The progeny continued under the same dietary conditions up to the F5 generation (Baosheng et al., 2024). This strategy facilitates a comprehensive examination of how dietary adenine impacts the gut microbiota over successive generations, highlighting potential transgenerational effects and maintaining uniform experimental protocols.

In the preliminary phase of this study, seven experimental groups were established: a control group, and groups treated with adenine concentrations of 0.025, 0.05, 0.075, 0.10, 0.15, and 0.20%. Each group consisted of 50 *Drosophila* eggs used to observe developmental timing. Results indicated that larvae in the 0.15% and 0.2% treatment groups died before pupating, leading to the exclusion of these two groups. To better explore the effects and trends of adenine in *Drosophila*, the final experimental setup consisted of three groups: 0%, 0.05%, and 0.10%.

In this study, W1118 wild-type *D. melanogaster*, owned and maintained by the uric acid physiological functions research team at Jiamusi University, were reared in a meticulously controlled incubator at a temperature of 25 ± 0.5 °C and a relative humidity of 40 ± 0.5% and subjected to a 12-hour photoperiod. Adenine was obtained from Suolaibao Biotechnology Co., Ltd. (Beijing, China).

After culturing one vial each of male and female *Drosophila* from the F1 generation for 15 days, 25 *Drosophila* from each group were collected and homogenized in a glass homogenizer using a weight (g) to PBS (phosphate-buffered saline) ratio of 1:9. Following centrifugation, the supernatant was collected. The uric acid content in the flies was measured using an ELISA kit, and the assay was repeated three times to ensure accuracy. The average uric acid level for each experimental group was then calculated and recorded (Yurong et al., 2022).

### 2.2 Sampling experiments

After 15 days of culture, specimens of *D. melanogaster* from each experimental group were anaesthetised using CO_2_ and briefly decontaminated with 70% ethanol. Specimen were then rinsed three times with 1 × PBS, and the abdomen was dissected under a stereomicroscope (Ott et al., 2021; Fuse et al., 2023; Baosheng et al., 2024). The dissected tissues were preserved in centrifuge tubes at −80°C.

### 2.3 DNA extraction and PCR amplification

The FastDNA^®^ Soil Kit (MP Biomedical, USA) manufacturer’s protocol was followed to extract microbial DNA from the gut microbiota by processing homogenized samples. DNA quality was assessed via 1% agarose gel electrophoresis and a NanoDrop 2000 spectrophotometer (Thermo Scientific, US) to determine the A260/A280 ratio and DNA concentration (Zhu et al., 2024). The primers 806R (5’-GGACTACHVGGGTWTCTAAT-3’) and 338F (5’-ACTCCTACGGGAGGCAGAG-3’) were used to amplify the V3-V4 region of the bacterial 16S rRNA gene. The PCR protocol was initiated with denaturation at 95°C for three minutes, followed by 27 cycles of denaturation for 30 seconds at 95°C, annealing for 30 seconds at 55°C, and extension for 45 seconds at 72°C, with a final extension lasting 10 minutes at 72°C. To assemble the PCR mixture, 4 μL of 5x FastPfu Buffer, 0.4 μL of FastPfu Polymerase, 2 μL of 2.5 mM dNTPs, 10 ng of template DNA, and 0.8 μL of each primer at 5 μM were combined in 20 μL of sterile double-distilled water (ddH_2_O). After separation via 2% agarose gel electrophoresis, the PCR products were subjected to purification with an AxyPrep DNA Gel Extraction Kit (Oxygen Biosciences), followed by DNA quantification via a QuantiFluor-ST™ Assay Kit (Promega) (Long et al., 2024).

### 2.4 Illumina MiSeq sequencing

After the amplicon concentrations were normalized, the samples were subjected to quality evaluation, quantification, and sequencing on the Illumina MiSeq platform (USA) via the paired-end 2 × 300 base pair (bp) read method (Long et al., 2024). The online Supplementary material provides additional details.

### 2.5 Processing of sequencing data

After demultiplexing, the initial fastq files underwent Trimmomatic-based quality filtration, which was then followed by consolidation via FLASH. For 300 bp fragments, trimming was applied at positions with average quality scores less than 20 within a 50 bp window, while fragments greater than 50 bp in length were preserved for subsequent analysis (Zhu et al., 2024). Overlaps of more than 10 bp were used for the assembly of sequences, and sections that could not be assembled were discarded. Sequences affected by errors in barcoding, primer mismatches, or nucleotide ambiguities were removed. Operational taxonomic units (OTUs) were assigned at a 97% similarity threshold with the aid of UPARSE 7.1, and chimeric sequences were subsequently refined via UCHIME. All 16S rRNA sequences were classified on the basis of the Silva (SSU138) database, with adherence to a confidence threshold of 70% (Long et al., 2024).

### 2.6 Ecological and statistical analysis

The uric acid content in the flies was measured using an ELISA kit, and the assay was repeated three times to ensure accuracy. To examine uric acid levels, one-way ANOVA was conducted to assess statistically significant differences in the means among the GC, GL, and GH groups in the F1 generation. For multiple comparisons of the means across these different experimental groups, a Tukey HSD test was used. We used mothur v.1.30.2^1^ to generate rarefaction curves and evaluate gut microbiota alpha diversity metrics, including the abundance-based coverage estimator (ACE), Chao, and Shannon indices (Tong et al., 2023). The examination of alpha diversity was performed via one-way ANOVA and Tukey-Kramer post hoc tests (Tong et al., 2020b). Only *P-*values of 0.05 are shown. The gut microbiome health index (GMHI) and microbial dysbiosis index (MDI) were analyzed via species-level taxonomic analysis with MetaPhlAn2, and graphics were generated via the R (vegan 2.4.3) package (Gunathilake et al., 2020; Gupta et al., 2020).

The use of R software (version 3.3.1) facilitated the generation of Venn diagrams, which elucidated the unique and shared OTUs among the samples (Shade and Handelsman, 2012). The calculation of beta diversity distance matrices was performed via the QIIME platform.^2^ Nonmetric multidimensional scaling (NMDS) analysis, principal coordinate analysis (PCoA), and graphical representation were performed via R (vegan package) (Lozupone and Knight, 2005). The impact of different adenine levels (GC, GL, and GH) on community clustering and dispersion was assessed via analysis of similarity (ANOSIM) and multivariate nonparametric variance analysis models (Adonis, with 999 permutations) for weighted UniFrac distances and Bray-Curtis dissimilarities on the basis of the OTU data (Caporaso et al., 2010).

R software (version 3.3.1) was used to precisely quantify the species dominance and relative abundance at the designated taxonomic levels, employing “tax_summary_a” data. We used the Wilcoxon rank sum test for assessing abundance variations across two cohorts, incorporating the Benjamini-Hochberg false discovery rate (FDR) correction to account for multiple comparisons. Differences were deemed significant when the adjusted *P* values fell below the 0.05 threshold. Using the “ggtern” and “ggplot2” packages, a ternary plot was created to depict the associations and distributions of the dominant species ( > 0.5% relative abundance in at least one sample) across the groups (Xie et al., 2023). Using linear discriminant analysis (LDA) effect size (LEfSe) (LDA > 2), we distinguished specific phyla, genera, and species by combining statistical significance with biological relevance (Lian et al., 2019).

BugBase, a microbiota analysis tool, was used to identify and visualize predominant phenotypic traits in the microbiota samples (Lucas et al., 2018). BugBase was used to normalize OTUs via precomputed files for estimated 16S rRNA gene copy numbers to evaluate the characteristics of the microbiota (Lucas et al., 2018). To assess the differences in relative abundance between the control group and experimental groups, we conducted the Kruskal-Wallis H test at a significance level of *P* < 0.05. Using phylogenetic investigation of communities by reconstruction of unobserved states (PICRUSt2) analysis, we compared the functional profiles of the microbiota in the GL, GC, and GH groups (Langille et al., 2013). By analyzing 16S rRNA sequences, Kyoto Encyclopedia of Genes and Genomes (KEGG) Orthology (KO) functionalities of the microbiota were predicted, and the OTUs were correlated with gene content through phylogenetic trees derived from this analysis (Langille et al., 2013). Predictions by PICRUSt2 hinge on phylogenetic tree architectures and nearest neighbour identifications and are effective even over extensive distances (Lucas et al., 2018).

## 3 Results

### 3.1 Adenine increases uric acid

**FIGURE 1:**
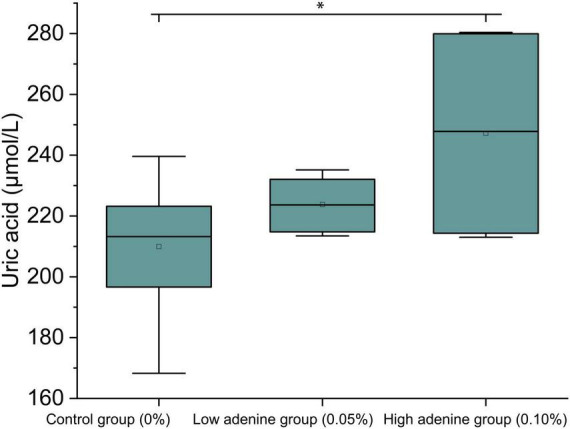
Adenine intake increased uric acid levels. Adenine intake significantly increased uric acid levels in the GH group (high adenine group) relative to those in the GC group (control group).

The uric acid levels significantly increased with adenine intake (df = 2, *F* = 7.52, *P* = 0.02; Figure 1). The GC group had the lowest median uric acid concentration, which was slightly greater in the GL group; and the GH group presented the highest uric acid levels, which were significantly different from those of the GC group (Tukey HSD, *P* = 0.01; Figure 1). This suggested that adenine intake might be associated with an increase in uric acid levels.

### 3.2 Gut microbiota health index and microbiota dysbiosis index

Illumina MiSeq sequencing revealed 3,613,244 high-quality sequences. These OTUs were classified on the basis of > 97% sequence identity, and 165 OTUs with an average length of 418 bp per read were obtained. Rarefaction and Shannon curves effectively depicted the sequencing depth (Supplementary Figure 1). The plateau status of the rarefaction curves suggested that the sequencing depth was sufficient (Supplementary Figure 1).

**FIGURE 2:**
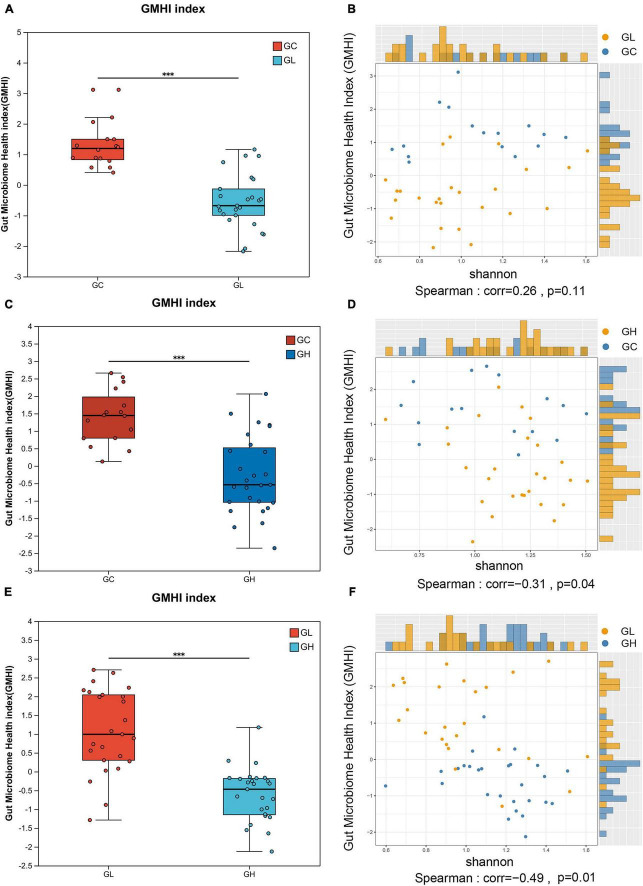
Analysis of differences in the gut microbiota health index (GMHI) of the *Drosophila melanogaster* gut microbiota and analysis of the consistency of alpha diversity after exposure to various adenine concentrations in the culture medium. **(A,C,E)** Demonstrate the significant differences between pairwise samples, with the x-axis representing group names and the y-axis indicating the index range for each group. *** Indicates *P* ≤ 0.001. In **(B,D,F)**, each point corresponds to a sample, with the y-axis representing the GMHI and the x-axis representing the alpha diversity index.

The GMHI indicated significant disparities in gut microbiome health across groups. Compared with the GH group, the GL group presented a significantly greater index value, and the GC group presented a greater index value than both the GL and GH groups did (Wilcoxon rank sum test, *P* < 0.05; Figures 2A, C, E). In the GC and GL groups, the GMHI was not significantly positively correlated with the Shannon index (*P* > 0.05; Figure 2B). In the GC and GH groups and in the GH and GL groups, the GMHI was negatively correlated with the Shannon index (*P* < 0.05; Figures 2D, F). Compared with the Shannon diversity, the GMHI performed significantly better in terms of stratification in the GC and GH groups and in the GH and GL groups (Figures 2D, F).

### 3.3 Alpha diversity and MDI

**FIGURE 3:**
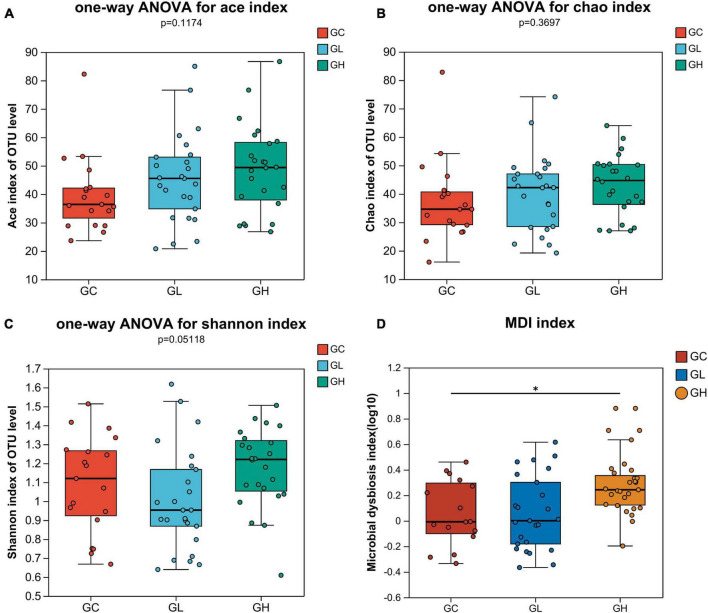
Alpha diversity comparison and analysis of the differences in the multidimensional deprivation indices of the gut microbiota of *Drosophila melanogaster* exposed to various adenine concentrations in the culture medium. The alpha diversity of *Drosophila* was measured by the ACE **(A)**, Chao **(B)**, and Shannon **(C)** indices. Alpha diversity analysis was performed via one-way ANOVA and Tukey-Kramer *post-hoc* tests. Higher values of the MDI **(D)** indicate greater disturbance of the microbiome. The graph shows significant differences among groups, with the *x*-axis representing group names and the *y*-axis showing the range of index values for each group. *Indicates 0.01 < *P* ≤ 0.05.

The ACE, Chao, and Shannon indices revealed that there were no significant differences in the gut microbiota diversity among the groups (one-way ANOVA, Tukey-Kramer, *P* > 0.05; Figures 3A–C). The MDI showed that gut microbiome dysbiosis significantly differed between the GC group and the GH group (Wilcoxon rank sum test, multiple test correction: FDR, *P* < 0.05; Figure 3D), but there were no significant differences between the GC and GH groups or between the GL and GH groups (Wilcoxon rank sum test, multiple test correction: FDR, *P* > 0.05; Figure 3D).

### 3.4 Beta diversity

**FIGURE 4:**
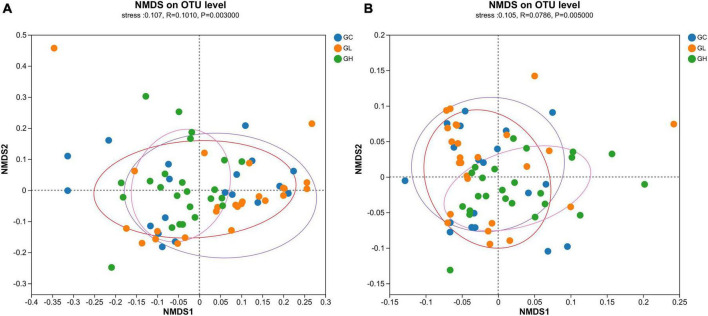
Assessment of the impact of various adenine concentrations in the culture medium on the microbial diversity of *Drosophila*. To investigate the effects of different uric acid levels (GC, GL, and GH) on community clustering and dispersion, the Bray-Curtis dissimilarity **(A)** and weighted UniFrac distance **(B)** were extracted via an OTU-level database. Each data point represented in the NMDS plots corresponds to an individual sample collected from the gut.

The gut microbiotas of the GC, GL and GH groups were significantly different, as indicated by the Bray-Curtis dissimilarity matrix (Adonis: R^2^ = 0.077, *P* = 0.019; ANOSIM, statistic = 0.101, *P* = 0.003; Figure 4A) and weighted UniFrac distances (Adonis: R^2^ = 0.078, *P* = 0.013; ANOSIM, statistic = 0.078, *P* = 0.005; Figure 4B).

No significant variations in the gut microbiota among the F1C, F1L, and F1H groups were detected by the Bray-Curtis distance metric (Adonis: *R*^2^ = 0.3714, *P* = 0.2860). The Bray-Curtis distance metric (Adonis: R^2^ = 0.2710, *P* = 0.0280; Supplementary Figure 2A) revealed notable variations in the gut microbiota among the F2C, F2L, and F2H groups. No significant variations in the gut microbiota among the F3C, F3L, and F3H groups were detected by the Bray-Curtis distance metric (Adonis: *R*^2^ = 0.1270, *P* = 0.9140; Supplementary Figure 2B). The Bray-Curtis distance metric (Adonis: *R*^2^ = 0.2097, *P* = 0.0310; Supplementary Figure 2C) revealed significant variability in the gut microbiota among the F4C, F4L, and F4H groups. The differences in the microbiota among the F5C, F5L, and F5H groups were statistically significant according to the Bray-Curtis distance metric (Adonis: *R*^2^ = 0.2639, *P* = 0.0190; Supplementary Figure 2D).

### 3.5 Gut microbiota composition

**FIGURE 5:**
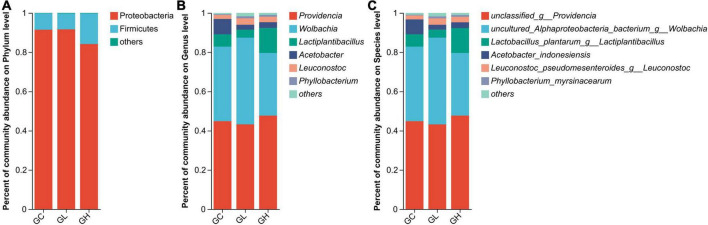
Comparison of the effects of various adenine concentrations in the culture medium on the gut microbiota composition of *Drosophila*. Bar graphs were generated to visualize the microbial communities present at the phylum **(A)**, genus **(B)**, and species **(C)** levels. The provided figures specifically depict phyla, genera, and species, showing those with relative abundances exceeding 1% in at least one sample.

The gut microbiota of the GC, GL and GH groups were dominated by Proteobacteria (91.45%, 91.65%, and 84.15%, respectively) and Firmicutes (8.34%, 8.08%, and 15.55%, respectively) (Figure 5A). In addition, *Providencia* (44.84%, 43.20%, and 47.68%), *Wolbachia* (38.03%, 44.20%, and 31.92%), *Lactiplantibacillus* (6.28%, 4.09%, and 12.70%), *Acetobacter* (7.79%, 2.52%, and 2.98%) and *Leuconostoc* (2.04%, 3.26%, and 2.81%) dominated the gut microbiota of the GC, GL and GH groups, respectively (Figure 5B).

In the GC, GL and GH groups, the gut microbiomes were chiefly composed of *unclassified_g__Providencia* (44.84%, 43.20%, and 47.68%, respectively), *uncultured_Alphaproteoba cteria_bacterium_g__Wolbachia* (38.03%, 44.20%, and 31.92%, respectively), *Lactobacillus_plantarum_g__Lactiplantibacillus* (6.28%, 4.09%, and 12.70%, respectively), *Acetobacter_indonesiensis* (7.55%, 2.50%, and 2.93%, respectively), and *Leuconostoc_pseudomesenteroides_g__Leuconostoc* (2.04%, 3.26%, and 22.81%, respectively) (Figure 5C).

### 3.6 Microbial diversity: trispecies overlap

*Providencia* (Proteobacteria) accounted for 45.2% of the total bacteria in the GC, GH, and GL groups, with relative abundances of 33.0%, 35.1%, and 31.8%, respectively (Supplementary Figure 3A). *Lactiplantibacillus* (Firmicutes) accounted for an average percentage of 7.7% of bacteria in the GC, GH, and GL groups, with respective relative abundances of 27.2%, 55.1%, and 17.7% (Supplementary Figure 3A). Across the GC, GH and GL groups, a shared set of 64 OTUs was identified (Supplementary Figure 3B). The GC group presented 39 unique OTUs, the GH group presented 10, and the GL group presented 25. A shared subset of 8 OTUs was observed between the GC and GH groups. A subset of 9 OTUs was observed to be shared between the GC and GL groups. The GH and GL groups shared a subset of 10 OTUs (Supplementary Figure 3B).

### 3.7 Differential microbiota compositions

**FIGURE 6:**
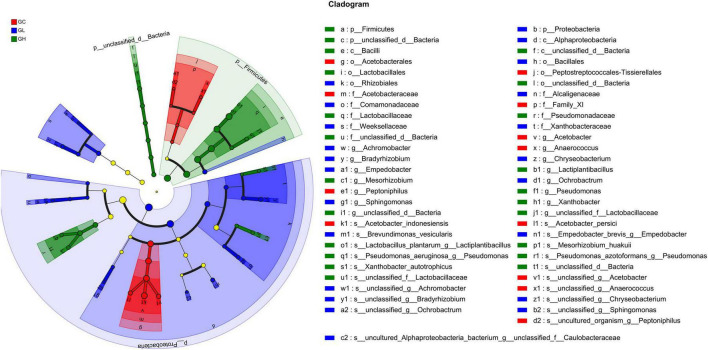
Linear discriminant analysis effect size (LEfSe) analysis of gut bacterial biomarkers in *Drosophila* treated with different adenine concentrations in the culture medium.

At the phylum level, LEfSe analysis revealed that Proteobacteria were enriched in the GL group, and Firmicutes and *unclassified_d__Bacteria* were enriched in the GH group (LDA > 2, *P* < 0.05; Figure 6). At the genus level, the GC group was enriched with *Acetobacter*, *Anaerococcus*, *and Peptoniphilus*; the GL group was enriched with *Achromobacter*, *Bradyrhizobium*, *Chryseobacterium*, *Empedobacter*, *Ochrobactrum*, and *Sphingomonas*; and the GH group was enriched with *Lactiplantibacillus*, *Pseudomonas*, *Mesorhizobium*, *unclassified_d__Bacteria*, *unclassified_f__Lactobacillaceae*, and *Xanthobacter* (LDA > 2, *P* < 0.05; Figure 6). At the species level, the GC group was enriched with *Acetobacter_persici*, *Acetobacter_indonesiensis*, *uncultured_organism_g__Peptoniphilus*, *unclassified_g__Acetobac ter*, and *unclassified_g__Anaerococcus*; the GL group was enriched with *Brevundimonas_vesicularis*, *Empedobacter_brevis_g__ Empedobacter*, *unclassified_g__Sphingomonas*, *unclassified_g__ Achromobacter*, *unclassified_g__Bradyrhizobium*, *unclassified_g__ Chryseobacterium*, *unclassified_g__Ochrobactrum*, and *uncultured_Alphaproteobacteria_bacterium_g__unclassified_f__ Caulobacteraceae*; and the GH group was enriched with *Lactobacillus_plantarum_g__Lactiplantibacillus*, *Mesorhizobium_ huakuii*, *Pseudomonas_aeruginosa_g__Pseudomonas*, *Pseudomonas_azotoformans_g__Pseudomonas*, *unclassified_d__ Bacteria*, *unclassified_f__Lactobacillaceae*, and *Xanthobacter_ autotrophicus* (LDA > 2, *P* < 0.05; Figure 6).

### 3.8 BugBase phenotype prediction and predicted functional analysis

In the gut microbiota, four phenotypes, namely, containing mobile elements, gram positivity, biofilm formation, and gram negativity, were significantly different among all the treatment groups (Kruskal-Wallis H test, *P* < 0.05; Supplementary Figure 4). There were no differences in the five phenotypes, namely, anaerobic, aerobic, facultatively anaerobic, potentially pathogenic, and stress tolerant, among the treatment groups (Kruskal–Wallis H test, *P* > 0.05; Supplementary Figure 4).

**FIGURE 7:**
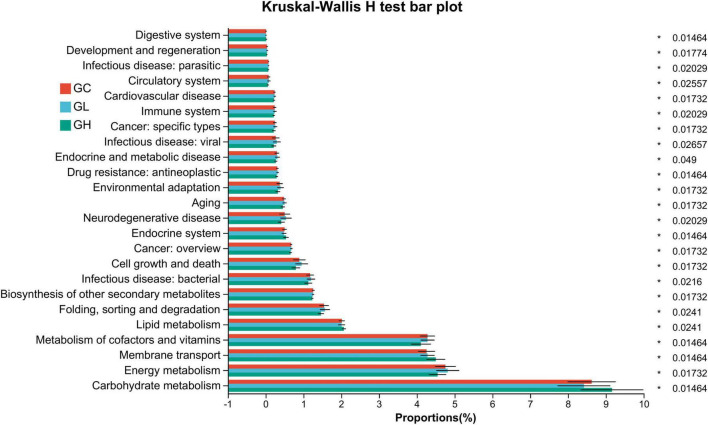
Predictive functional profiling of the *Drosophila* gut microbiota via phylogenetic investigation of communities via reconstruction of unobserved states. Significant variation was observed in the relative abundance of the predicted genes linked to secondary KEGG pathways within the metagenomic data. The accompanying list delineates the prevalence of each functional pathway alongside its corresponding secondary KEGG pathway. The color-coding scheme represents the differences in the gut microbiota between the control and adenine-treated groups: red represents the GC group, blue represents the GL group, and green represents the GH group.

In the gut microbiota, 24 KEGG pathways showed significant differences: aging, biosynthesis of other secondary metabolites, cardiovascular disease, circulatory system, cancer: specific types, carbohydrate metabolism, cancer: overview, cell growth and death, drug resistance: antineoplastic, digestive system, development and regeneration, energy metabolism, endocrine and metabolic disease, environmental adaptation, endocrine system, folding, sorting and degradation, infectious disease: bacterial, infectious disease: parasitic, infectious disease: viral, immune system, lipid metabolism, metabolism of cofactors and vitamins, neurodegenerative disease, and membrane transport (Kruskal–Wallis H test with FDR correction, *P* < 0.05; Figure 7).

## 4 Discussion

In this study, we observed that there were no significant differences in the alpha diversity of the gut microbiota of *D. melanogaster* that were administered different concentrations of adenine. However, significant differences were found in the MDI and GMHI. This finding reveals that traditional diversity indices may not be sufficient to fully reflect the complexity and health status of the gut microbiota (Gunathilake et al., 2020). The MDI clearly differed between the GC and GH groups, indicating that a high-purine diet may lead to a significant imbalance in the gut microbial community (Gupta et al., 2020). This imbalance is likely related to the accumulation of purine metabolites in the gut microbiota, which can affect microbial metabolism and interactions, potentially leading to dysbiosis and health issues for the host (Yamauchi et al., 2020). Conversely, the GMHI reflected significant differences in the gut microbiota among groups, with the GL group having a healthier microbial composition than the GH and GC groups did (Li X. et al., 2022). This suggests that reducing purine intake could enhance microbial health, leading to a more balanced gut microbiota and improved host health. Understanding the mechanisms by which purine intake affects the gut microbiota composition could inform dietary recommendations and therapeutic strategies to mitigate purine-related health risks (Wong et al., 2014). Future research should investigate the specific microbial species involved and their metabolic pathways affected by purine levels, providing deeper insights into how diet shapes the gut microbiota and health of the host.

The correlation between the GMHI and Shannon index differed between the low- and high-purine diet groups, reflecting complex alterations in the structure and function of the gut microbiota under varying dietary conditions (McMullen, 2020). Interestingly, the traditional alpha diversity index revealed no significant differences among groups, indicating that despite unchanged microbial species diversity and evenness, the health and balance of these communities are affected by dietary changes (Wesseltoft et al., 2024). This finding highlights the limitations of alpha diversity in assessing the health impacts of the microbiome, emphasizing the value of functional health indices such as the GMHI and MDI (Buchon et al., 2013). These insights suggest that we could strategically manipulate gut microbiome health through dietary modifications, particularly changes in the purine content, which could inform dietary guidelines and therapeutic strategies for microbiome-associated diseases. Organ meats, some seafood, and legumes should be reduced to decrease uric acid and promote the gut microbiota. An increase in low-purine foods (dairy, fruits, and vegetables) and prebiotic and probiotic foods (fiber-rich vegetables and fermented items) promotes beneficial bacteria and gut barrier integrity. Personalized diets may prevent or treat microbiome problems.

Our findings show that increased purine concentrations significantly alter the gut microbiota structure in *Drosophila*. As adenine levels increase, uric acid levels also increase, affecting the composition and function of the gut microbiota (van Dam et al., 2020; Yamauchi et al., 2020). Elevated uric acid levels modify the gut pH and biochemical parameters, favoring specific microbial species (Engel and Moran, 2013). *Lactobacillus* and *Bifidobacterium* thrive in high uric acid environments by metabolizing uric acid, reducing systemic uric acid levels (Nesse, 2011; Jia et al., 2024). Elevated uric acid is linked to inflammatory conditions such as gout and cardiovascular diseases, which can alter the immune response or gut barrier function, influencing microbiota architecture (Lv et al., 2020; Wang Z. et al., 2022). Interactions among the gut microbiota can lead to shifts in community structure (Lozupone et al., 2012). Competition for resources allows specific microbiota to adapt better to the host environment, inducing changes in the overall microbiota (Macke et al., 2017). For example, *Lactobacillus* reduces the gut pH through lactic acid production, excluding most yeasts and thus promoting the growth of *Acetobacter* and other acid-tolerant bacterial populations (Ansari et al., 2023). These dynamic alterations ultimately affect the overall balance and health status of the *Drosophila* gut microbiota (Erkosar and Leulier, 2014). Long-term coevolution optimizes host-microbiota adjustments, meeting host physiological demands (Yang, 2020). *Lactobacillus* can facilitate nutrient absorption by breaking down yeast polysaccharides (Storelli et al., 2018). These findings highlight the mechanisms by which metabolic products such as purines influence host health via impacts on the gut microbial balance, providing a scientific basis for future exploration of interactions between purine metabolism and the gut microbiota (Jia et al., 2023).

Purine addition to the diet may directly impact gut microbial ecology (Kasahara et al., 2023). Because purines are a component of nucleic acids, increasing the purine content in *D. melanogaster* diets may alter intestinal dietary constituent ratios (Livelo et al., 2023). Specific microbial species utilize purines as metabolic substrates, potentially promoting targeted bacterial growth (Liu et al., 2020). Gut microbes engage in mutualistic and competitive interactions. Adding purines to the diet may alter these interactions, potentially affecting microbial community composition and function (Wang et al., 2024). Adding purines to the diet could modify the *Drosophila* gut microbiota by changing the intestinal chemical environment, nourishing specific microbes, and affecting the innate immune response of the host (Liu et al., 2023). These findings illuminate the link between gut health and purine intake, offering insights into comparable mechanisms in higher organisms (Kasahara et al., 2023).

The use of *D. melanogaster* as a model organism offers numerous advantages for genetic and metabolic studies because of its genetic simplicity, short life cycle, and ease of laboratory manipulation (Fischer et al., 2023; Rand et al., 2023). However, compared with humans, mice (*Mus musculus*) and zebrafish (*Danio rerio*) also offer model organism advantages but have limitations, with mice being closer physiologically to humans and zebrafish benefiting from rapid development and transparent embryos. The extrapolation of these findings to humans must be approached with caution due to significant biological differences. Physiologically, the renal system of *Drosophila*, the Malpighian tubules, operates differently from that of human kidneys and plays a crucial role in uric acid regulation (Orchard et al., 2023). This distinction exemplifies broader physiological and anatomical divergences that can affect the extrapolation of metabolic and excretory processes. Additionally, the complexity of human metabolism, with its multiple redundant pathways and diverse microbiota influenced by a wide range of factors, adds layers of complexity that are absent in *Drosophila*. The human immune system is also significantly more complex, particularly in terms of interactions with the microbiota (Tafesh-Edwards and Eleftherianos, 2023). Furthermore, the generational and long-term effects observed in human studies span much longer periods, making direct comparisons with *Drosophila* challenging (Yang et al., 2020). The differences in metabolic rates and body sizes between humans and *Drosophila* also affect the scale and nature of their biological responses to dietary changes, underscoring the need for the cautious application of *Drosophila* research findings to human health contexts.

Changes in diet, specifically increased purine intake, lead to significant alterations in the *Drosophila* intestinal microbiota (Yamauchi et al., 2020). LEfSe analysis at the genus level indicated significant enrichment of *Acetobacter*, *Pseudomonas*, and *Sphingomonas* across the different experimental groups. These genera are crucial for the metabolism of uric acid, a byproduct of purine metabolism (Bratty et al., 2011). *Acetobacter* was enriched in the GL group and may reduce uric acid levels by metabolizing ethanol and other organic acids, indirectly influencing uric acid dynamics (Li W. et al., 2022). *Pseudomona*s, enriched in the GH group, adapts to hyperuricaemic conditions, increasing its ability to detoxify uric acid (Bai et al., 2023). *Sphingomonas* was enriched in the GL group. *Sphingomonas*, enriched in the GL group, may aid in degrading uric acid or its precursors, maintaining metabolic homeostasis in the gut (Saati-Santamaría et al., 2021). Recent studies have suggested that the metabolic activities of these bacteria are pivotal for managing uric acid levels and for influencing systemic metabolic processes, potentially offering novel insights into therapeutic strategies for managing diseases such as gout (Mondal et al., 2023). The interplay between the microbiota and its host results in a sophisticated microbial network that is dynamically responsive to dietary inputs (Kolodziejczyk et al., 2019). The ability of these genera to adapt to elevated uric acid levels suggests a complex evolutionary advantage that may have significant implications for host health (Winans et al., 2017). Understanding the interactions between these microbes and the host, particularly in the context of diet-induced changes, could provide valuable insights into their development (Capo et al., 2019).

This study revealed significant differences in 24 KEGG pathways in the *Drosophila* gut microbiota due to varying dietary adenine concentrations, demonstrating a complex interplay between diet, microbial response, and host metabolic pathways (Zhang et al., 2022). By modulating adenine concentrations, we found that even minor dietary changes can significantly alter microbial dynamics and, by extension, host health (Capo et al., 2019). Notably, pathways involved in ageing, cancer, cardiovascular diseases, and cofactor and vitamin metabolism showed considerable shifts, highlighting the potential of microbial metabolism to directly influence host energy production and fat storage, which are crucial factors in health maintenance and disease prevention (Tran and Mohajeri, 2021). Alterations in pathways related to cancer, cardiovascular, and neurodegenerative diseases suggest significant long-term effects of microbiota changes on host disease susceptibility (Sorboni et al., 2022). This aspect is critical because it links dietary components and microbiota-mediated metabolic changes to specific health outcomes, aligning with findings from other studies that suggest that dietary influences on the microbiota can directly impact host disease pathways (Haran and McCormick, 2021). The observed changes in immune and infectious disease pathways reinforce the role of the gut microbiota in host defense (Tafesh-Edwards and Eleftherianos, 2023). The adaptability of the gut microbiota to dietary shifts, which are crucial for modifying pathways and maintaining homeostasis, is vital in changing environmental conditions (Guo et al., 2024). Future studies should explore the molecular mechanisms by which diet-induced microbiota alterations influence host health (Lee et al., 2020). Specifically, research could focus on the reversibility of pathway changes with dietary interventions, potentially leading to novel dietary guidelines and therapeutic strategies (Wu et al., 2022). Additionally, longitudinal studies on the persistence of dietary effects on the microbiota across host life stages could yield insights into the optimal timing and permanence of interventions (Sorboni et al., 2022).

## 5 Conclusions

In conclusion, our study demonstrated that dietary adenine significantly impacts the composition of the gut microbiota across multiple generations of *D. melanogaster*. We observed notable dysbiosis in the gut microbiome, particularly in the low- and high-adenine groups, indicating a direct correlation between adenine intake and the microbial community structure. This dysbiosis coincided with increased uric acid levels, suggesting a potential link among dietary adenine, gut microbiota alterations, and metabolic health. Our findings underscore the importance of understanding the intricate interplay among dietary components, the gut microbiota, and metabolic processes, especially in the context of diseases such as HUA. Furthermore, the use of *Drosophila* as a model organism offers valuable insights into the long-term effects of dietary interventions on the microbiome and metabolic health. Future research should further explore the specific mechanisms underlying these interactions to develop targeted strategies for preventing and treating metabolic diseases.

## Data availability statement

The authors acknowledge that the data presented in this study must be deposited and made publicly available in an acceptable repository, prior to publication. Frontiers cannot accept a manuscript that does not adhere to our open data policies.

## Ethics statement

The animal study was approved by the Jiamusi University’s Institutional Animal Care and Use Committee (IACUC). The study was conducted in accordance with the local legislation and institutional requirements.

## Author contributions

XY: Writing−review and editing, Writing−original draft, Supervision, Software, Resources, Methodology, Investigation, Formal analysis. QT: Writing−review and editing, Software, Data curation. JWa: Writing−original draft, Software, Resources, Investigation, Funding acquisition. JW: Writing−review and editing, Software, Data curation. ZQ: Writing−review and editing, Data curation. YW: Writing−review and editing, Data curation. RZ: Writing−review and editing, Data curation. BG: Writing−review and editing, Investigation. HQ: Writing−review and editing, Visualization, Supervision, Resources, Funding acquisition, Conceptualization.

## Footnotes

1
https://mothur.org/wiki/calculators/

2
http://qiime.org/scripts/assign_taxonomy
