# Supplementary material for: The impact of altered dietary adenine concentrations on the gut microbiota in Drosophila
Source: Front Microbiol. 2024 Aug 5;15:1433155. doi: 10.3389/fmicb.2024.1433155 (PMC11330887; doi:10.3389/fmicb.2024.1433155)
Supplement: Supplementary file 1 [file Data_Sheet_1.docx]

**Supplementary Material**

**The Impact of Altered Dietary Adenine Concentrations on the Gut Microbiota in *Drosophila***

Xianglin Yin^1, 2^, Qing Tong^3^, Jingtao Wang^1^, Jinfeng Wei^2^, Zhenbo Qin^2^, Yujie Wu^2^, Ruidi Zhang^2^, Baosheng Guan^2^, Hongbin Qiu^1, 2*^

1. School of Basic Medical Sciences, Jiamusi University, Heilongjiang, China;

2. School of Public Health, Jiamusi University, Heilongjiang, China;

3. School of Biology and Agriculture, Jiamusi University, Heilongjiang, China.

*Corresponding author

Hongbin Qiu

Tel.: +86-454-8603923

Fax: +86-454-8603923

E-mail: qiuhongbin63@163.com

Word count: 6660

Number of figures: 7


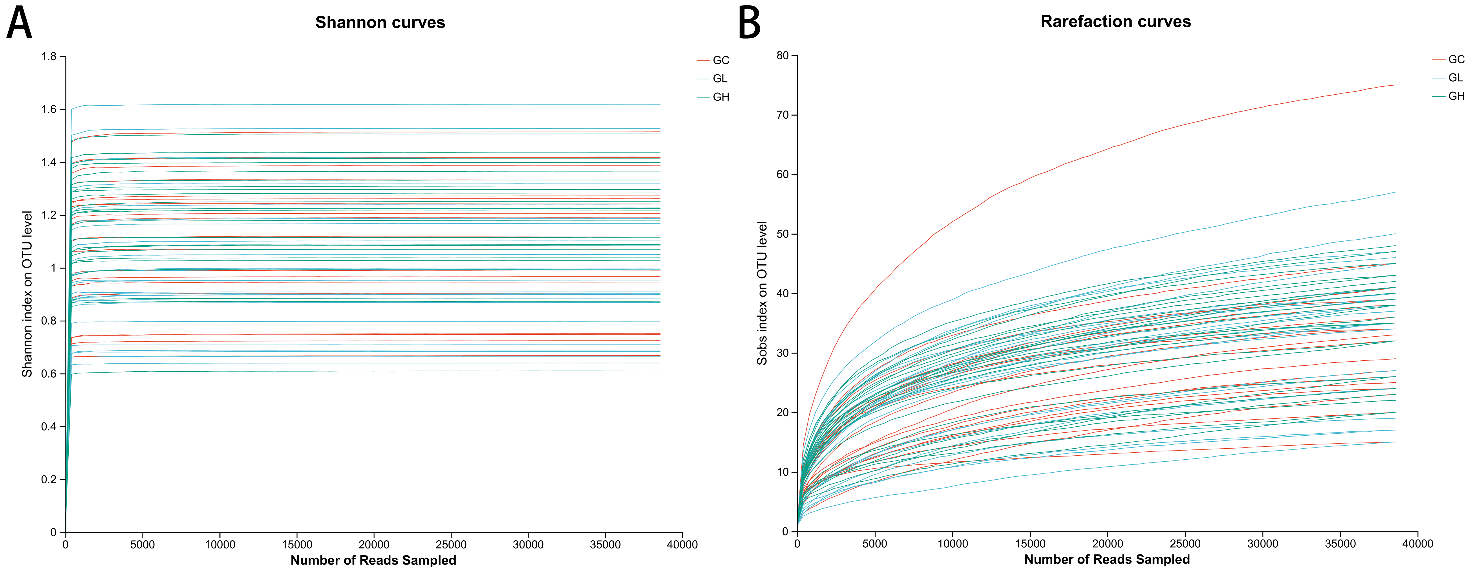


**Figure S1** Shannon curves and Rarefaction curves.

The Shannon curves and Rarefaction curves were plots of the number of OTUs as a function of the number of sequences. The Shannon curves reflect the microbial diversity of the samples at different sequencing quantities.


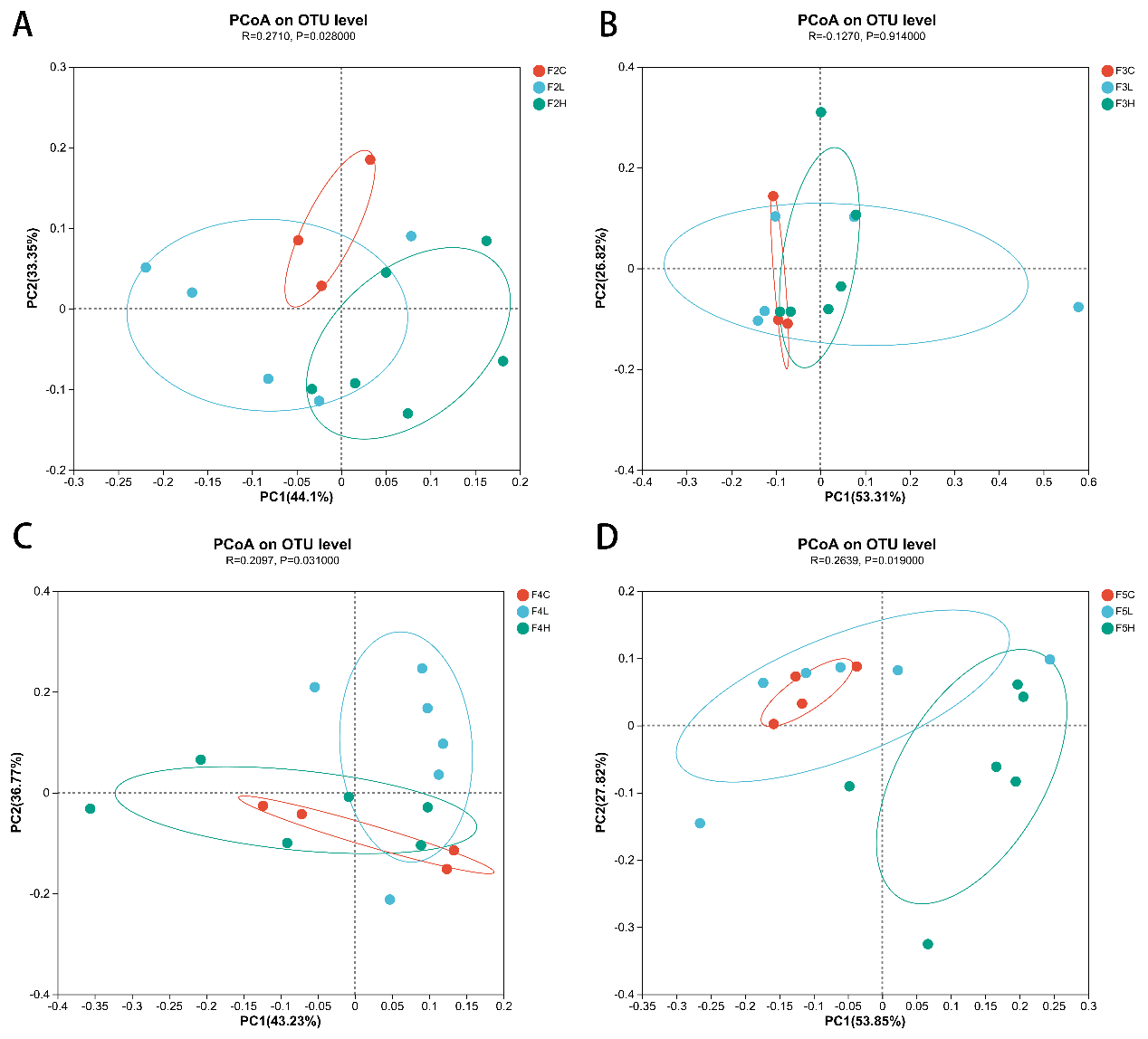


**Figure S2** PCoA Analysis of Microbiota Structure Responses to Concentration Gradient Treatments Across Generations

This principal coordinate analysis (PCoA) plots unveils the diversity patterns within microbiota across successive generations (F2 to F5), contrasting control groups (C) with groups exposed to varying concentrations (low concentration group H, high concentration group L). Each plot (A to D) represents the spatial distribution of microbial operational taxonomic units (OTUs) at a specific generational level. The color of the points indicates the different treatment groups: red points represent the control group (FC), green indicates the high concentration exposure group (FL), and blue represents the low concentration exposure group (FH). The elliptical lines depict the spatial clustering trend within the same treatment group. R and P values within each plot quantify the strength and statistical significance of intergroup differences.


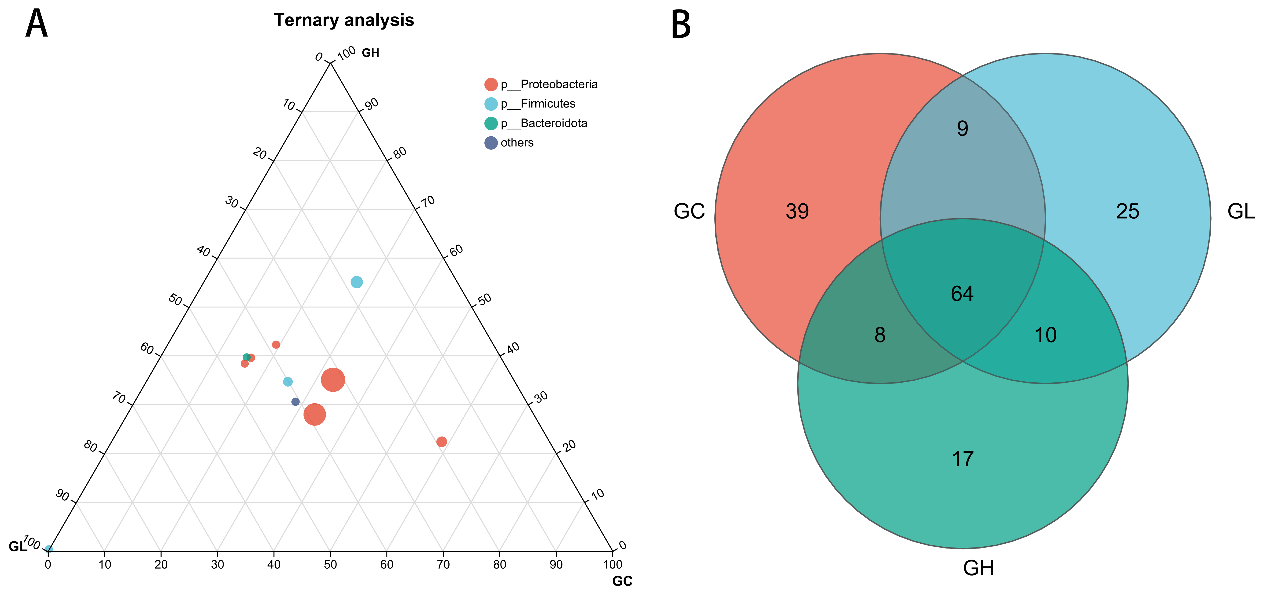


**Figure S3** A comparative Analysis of Microbiota: Ternary Plot and Venn Diagram.

The ternary diagram (A) illustrates the proportional interrelations of GH, GC, and GL. The plot points, differentiated by color, represent various bacterial phyla: Proteobacteria (red) and Firmicutes (blue), illustrating their relative abundances in each group. Each plot point's size reflects the abundance of its respective phylum. The Venn diagram (B), depicts the unique and shared species across three groups: GH, GC, and GL. The Venn diagram quantifies biodiversity and microbial species overlaps among the groups, with intersecting numbers indicating shared species and non-overlapping numbers showing unique species.**
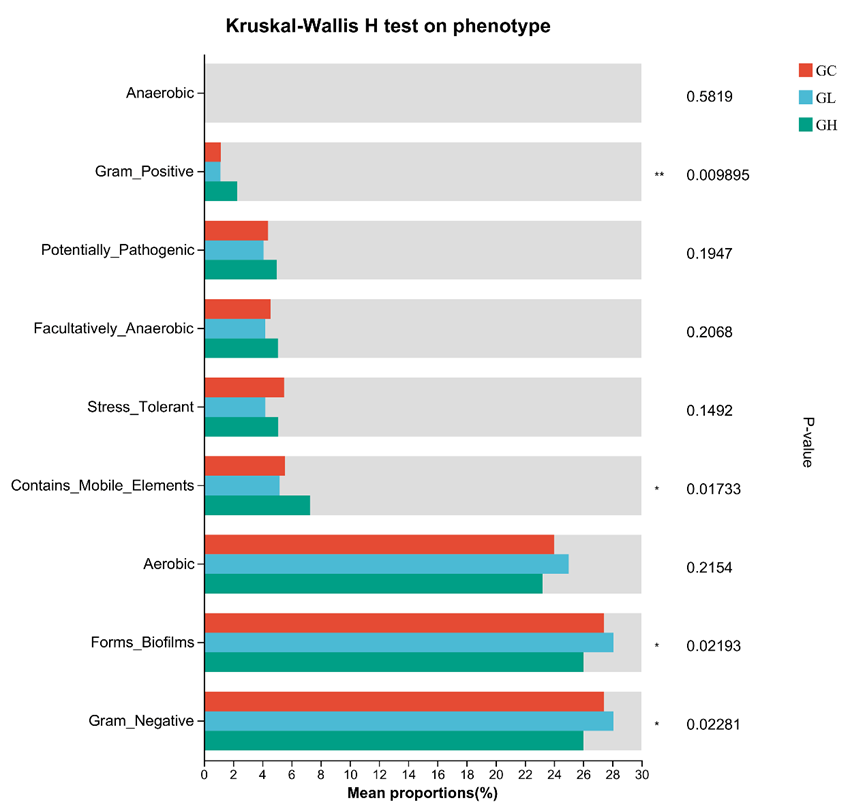
**

**Figure S4** Predictive functional profiling of *Drosophila* gut microbiota via BugBase.

The color-coding method illustrates the variations in skin and gut microbiota between the control group and the wildfire ash groups. Significance denoted by * and ** corresponds to 0.01 < *P* < 0.05 and 0.001 < *P* < 0.01respectively.
